# Does category of strength predict return-to-work after occupational injury?

**DOI:** 10.1186/s12889-022-13817-2

**Published:** 2022-08-02

**Authors:** Chia-Lin Yang, Yan-Ru Yin, Chuan-Man Chu, Pei-Ling Tang

**Affiliations:** 1 Department of Physical Medicine and Rehabilitation, Kaohsiung Veterans General Hospital, 386, Dazhong 1st Rd., Zuoying Dist., Kaohsiung City, 813414 Taiwan (ROC); 2 Department of Occupational Medicine, Kaohsiung Veterans General Hospital, 386, Dazhong 1st Rd., Zuoying Dist., Kaohsiung City, 813414 Taiwan (ROC); 3 Research Center of Medical Informatics, Kaohsiung Veterans General Hospital, 386, Dazhong 1st Rd., Zuoying Dist., Kaohsiung City, 813414 Taiwan (ROC); 4 Department of Health-Business Administration, Fooyin University, 151 Jinxue Rd., Daliao Dist., Kaohsiung City, 83102 Taiwan (ROC); 5 College of Nursing, Kaohsiung Medical University, 100, Shin-Chuan 1st Road, Sanmin Dist. Kaohsiung City, 80708 Taiwan (ROC)

**Keywords:** Carrying, Lifting, Physical capacity evaluation, Occupational rehabilitation, Return to work, Strength

## Abstract

**Background:**

Occupational accidents may lead laborers to lose their working capacities, affecting their physical and mental health. Occupational rehabilitation helps improve the ability of patients with occupational accidents and suggests appropriate jobs to avoid second injuries. This study aimed to identify whether any of the functional capacity evaluation (FCE) strength subtests predicted successful return to work.

**Methods:**

Data were collected of 84 patients receiving government-subsidized occupational rehabilitation between September 2016 and December 2018. A structured questionnaire was employed for pre- and post-training assessment, including basic information, information of the occupational accident, status of the laborer at the opening of the injury case, physical requirement for the job, and physical capacity. Eight subtests of strength were included in the physical capacity evaluation, i.e., carrying, lifting to several levels, power grip, and lateral pinch, to explore the association between the strength tests and return to work.

**Results:**

The unadjusted model showed that for every additional kilogram in bilateral carrying strength before work hardening training, the odds of successful return to work increased (crude odds ratio [OR] = 1.12, 95% confidence interval [CI] = 1.01–1.24, *p* = 0.027). After adjustment for basic demographic information and pre-accident physical functional elements of work, the odds of successful return to work increased (adjusted OR = 1.27, 95% CI = 1.04–1.54, *p* = 0.02) for every additional kilogram in the pre-training bilateral carrying strength. There were no statistically significant differences observed in the other seven subtests.

**Conclusion:**

Through thorough evaluation and work hardening training provided in the occupational rehabilitation, patients’ physical capacity can be understood and improved. However, a full evaluation of functional capacities is prolonged and time-consuming. This study provides evidence that pre-work-hardening bilateral carrying strength may be a promising predictor of return to work and we recommend to consider it as a prioritized test to assist in determining appropriate advice regarding return to work.

**Supplementary Information:**

The online version contains supplementary material available at 10.1186/s12889-022-13817-2.

## Background

Occupational accidents cause financial losses, impact physical and mental health of the affected, and sometimes cost workers their jobs. Work-related injuries, or traumas during the work, constitute a large type of occupational accidents, with the other type being work-related illnesses [1]. Work-related musculoskeletal disorders (WMSDs) are the most prevalent occupational diseases in Europe and the United States (US) [2], whereas occupational injuries are the most prevalent in Taiwan, mostly resulting from traffic accidents on the way to or from work, piercing injuries due to improper operation, and crushing injuries caused by falling objects [3]. Under the Labor Occupational Accident Insurance and Protection Act, the government in Taiwan provides financial and livelihood aids and occupational rehabilitation for workers with occupational accidents [4]. Occupational rehabilitation includes functional capacity evaluation (FCE), work hardening, psychological counseling and job accommodation, and is primarily aimed to enhance the work-related physical capacity of the injured for them to return to work (RTW) [5–7].

An inter-play of physical, psychological and social factors decides whether an injured individual can return to work unobstructed [8, 9], such as sex, age and education in the demographic elements, as well as accident details such as the accident nature, affected body area, disability, reported pain intensity and job type such as blue- vs. white-collar [8, 10–12]. Therefore, when an individual’s physical capacity is consistent with the job to be returned to and the requirements of the work setting, an optimal relationship between the individual, the setting and factional capacity is formed, increasing the probability for successful RTW [13].

By determining post-accident physical capacity and strength, the FCE plays a vital role in occupational rehabilitation and RTW planning [9, 14–17]. The FCE referred to in this study was designed based on the physical functional elements of work in the US Dictionary of Occupational Titles (DOT), and measures a variety of work-related physical capacities such as strength, mobility, hand coordination and position tolerance [5]. Studies have shown that FCE has a good inter-rater reliability; and for the bilateral carrying and lifting subtests, in particular, the inter-rater reliablity was 0.95–0.98 [18–21]. A few studies have already explored the strength subtests as predictors of return to work, but no consistent conclusion has been reached [9, 16]. More importantly, a full FCE takes about six to 7 h, which is a heavy burdern to healthcare professionals who are always busy. We therefore aimed to identify representative subtests from the strength subtests as significant predictive factors for return-to-work to provide guidance for heathcare professionals’ reference regarding laborers suffering occupational accidents in the clinical setting.

## Methods

### Data collection

This was a retrospective study that included subjects who had received government-subsidized occupational rehabilitation between September 2016 and December 2018. In the government subsidize rehabilitation program, patients with occupational accidents in the Occupational Medicine were transferred by the nurses there to the Occupational Rehabilitation Center as potential participants. At the Center, the staff screened the potential participants and interviewed the eligible ones, followed by visits for confirmation. The inclusion criteria included presence of occupational accidents, stable medical conditions, and willingness to RTW with clear RTW goals. A total of 139 individuals with occupational accidents were interviewed initially, among whom 84 met the inclusion criteria. After pre-training FCE, the participants received work hardening training twice week, with 2–3 hours each session, for an average period of 2 months. After the completion of the training, the participants received the post-rehabilitation FCE. They were followed up by phone on the RTW status 6 months after the completion of the training.

In this study, we used the information of the participants described above. As all participants signed a relevant Informed Consent Form (ICF) before they started rehabilitation in the government-subsidized program, the Internal Review Board (IRB) of Kaohsiung Veterans General Hospital, decided that this study would have no impact on the subjects and therefore waived collection of further ICF (IRB# VGHKS19-CT3–12).

### Measures

The initial interview questionnaire was a structured questionnaire developed by the research team. The questionnaire consisted of seven parts: basic information, description of occupational accidents, current medical situation, current employment status and disability identification, family and financial status of the worker, evaluation of mental health and family impact, work history and occupational skills, and functional evaluation. Each participant was interviewed in person and asked to describe his/her pre-accident work setting and requirements, including lifting, carrying, climbing, stooping-crouching, walking and repetitive sitting-standing, as shown in Additional file 1, Tables S1. Using this information, his/her pre-accident workload was classified into one of the following five categories: sedentary, mild load, moderate load, heavy load and very heavy load [22]. Considering the sample size of this study and the common injury types, we further grouped the five classifications of workload into two types based on the white- and blue-collar occupations, i.e., mild load (low physical demands) including sedentary and a light load vs. moderate load (high physical demands) including moderate, heavy and very heavy loads [23].

FCE was conducted after the initial interview, covering sensory function, range of motion (ROM), manual muscle testing (MMT), a 3-minute stepping test, physical fitness, and physical capacity evaluation. Physical capacity evaluation was carried out in four dimensions, one of which was “strength” that had eight subtests, including bilateral carrying, three types of bilateral lifting (floor to knuckle lifting, knuckle to shoulder lifting, and shoulder to overhead lifting), power grip (left and right), and lateral pinch (left and right). Not only were the maximum weights (kilograms) obtained for the eight strength subtests, but the weights were further categorized into the five load types (see Additional file 1, Tables S2). This study defined RTW as returning to the original job position and investigated the relationship between strength subtests and RTW.

### Statistical analysis

The IBM SPSS Statistics version 22.0 (SPSS, Inc., Chicago, IL, USA) was used for data processing after data collection. Continuous values were presented as mean ± standard deviation (SD), and the independent sample t-test was used to analyze continuous variables. The distribution of categorical variables was presented as samples and percentages, and the chi-square test or Fisher’s exact test was used to explore the relationship with RTW. The statistical results were presented in figures to assist in the description of the study. An unadjusted logistic regression analysis was first conducted to examine univariate associations between results of the carrying, lifting, power grip and lateral pinch subtests and RTW before and after work hardening training. In addition, associations between pre-post training differences in each of these measures and RTW was also examined. Then a multivariate logistic regression analysis was performed with different variables included to test whether an independent factor predicted RTW. The demographic factors (age, gender, marital status, education, and injury site) and pre-accident physical functional elements of work (pain, carrying, lifting, climbing, stooping-crouching, repetitive sitting-standing, and walking) were included in the model sequentially. The significance level α was established at 0.05, and *p* < 0.05 indicated statistical significance.

## Results

Among the 84 workers with occupational accidents examined in this study, 69 (82.1%) successfully returned to their original positions; their mean age was 41.24 years old, 64.3% were male; 58.3% were married, most had a high-school education or below (58.0%), 92.9% had occupational injuries, most had upper limb injuries (61.9%), and only three (3.6%) people with disability card (Table 1). The study revealed that 73.2% of the participants experienced pain. The pre-accident status of the six physical functional elements at work was as follows: most of the participants had moderate loads (including moderate, heavy and very heavy loads) for lifting, carrying, stooping-crouching, sitting-standing and walking, accounting for 78.0, 72.8, 76.8, 56.4 and 63.8%, respectively, but most had mild loads (including sedentary and light load) for climbing (65.4%) (Table 2).

**Table 1:** Effect of demographic characteristics on return-to-work

Characteristics		Return to original work		
	Total	Succeeded	Failed	***p***-value
	(***n*** = 84)	***n*** = 69 (82.1)	***n*** = 15 (17.9)	
	**n (%)**	**n (%)**	**n (%)**	
**Age** (y), Mean ± SD	41.24 ± 12.33	40.86 ± 12.08	43.00 ± 13.72	0.545 ^a^
**Sex**				0.702 ^b^
Male	54 (64.3)	45 (65.2)	9 (60.0)	
Female	30 (35.7)	24 (34.8)	6 (40.0)	
**Married**				0.885 ^b^
Yes	49 (58.3)	40 (58.0)	9 (60.0)	
No	35 (41.7)	29 (42.0)	6 (40.0)	
**Education**				0.117 ^b^
High school degree or below	47 (58.0)	41 (62.1)	6 (40.0)	
Bachelor’s degree or higher	34 (42.0)	25 (37.9)	9 (60.0)	
**Types of Occupational accidents**				0.290 ^c^
Work-related injury	78 (92.9)	65 (94.2)	13 (86.7)	
Work-related illness	6 (7.1)	4 (5.8)	2 (13.3)	
**People with Disability Card** ^**d**^				0.081^c^
Yes	81 (96.4)	68 (98.6)	13 (86.7)	
No	3 (3.6)	1 (1.4)	2 (13.3)	
**Injured limb**				0.168 ^b^
Upper limb	52 (61.9)	43 (62.3)	9 (60.0)	
Lower limb	11 (13.1)	7 (10.1)	4 (26.7)	
Other	21 (25.)	19 (27.5)	2 (13.3)	

*Note*: The numbers do not add up to 100% as some subjects did not complete some of the carrying or lifting tasks

^a^ Independent Sample t test

^b^ Chi-square test

^c^ Fisher’s exact test

^d^ People with Disability Card, which is a card issued by the Taiwanese government to eligible people with disability

**Table 2:** Effect of physical functional elements of work on return-to-work

		Return to original work		
Characteristics	Total	Succeeded	Failed	***p***-value ^a^
	(***n*** = 84)	***n*** = 69 (82.1)	***n*** = 15 (17.9)	
	n (%)	n (%)	n (%)	
**Pain**				0.213
Yes	60 (73.2)	51 (76.1)	9 (60.0)	
No	22 (26.8)	16 (23.9)	6 (40.0)	
**Lifting**				1.000
Mild load	18 (22.0)	15 (21.7)	3 (23.1)	
Moderate load	64 (78.0)	54 (78.3)	10 (76.9)	
**Carrying**				0.170
Mild load	22 (27.2)	16 (23.5)	6 (46.2)	
Moderate load	59 (72.8)	52 (76.5)	7 (53.8)	
**Climbing**				0.529
Mild load	53 (65.4)	44 (63.8)	9 (75.0)	
Moderate load	28 (34.6)	25 (36.2)	3 (25.0)	
**Stooping-Crouching**				1.000
Mild load	19 (23.2)	16 (23.2)	3 (23.1)	
Moderate load	63 (76.8)	53 (76.8)	10 (76.9)	
**Sitting-Standing**				0.195
Mild load	34 (43.6)	27 (40.3)	7 (63.6)	
Moderate load	44 (56.4)	40 (59.7)	4 (36.4)	
**Walk**				1.000
Mild load	29 (36.3)	25 (36.8)	4 (33.3)	
Moderate load	51 (63.8)	43 (63.2)	8 (66.7)	

*Note*. Physical functional elements pertain to pre-accident job requirements

Physical functional elements load classification: Mild load, including sedentary and light loads; moderate load, including moderate, heavy, and very heavy loads

The numbers do not add up to 100% as some subjects did not complete some of the carrying or lifting tasks

^a^ Fisher’s exact test

**Fig. 1:**
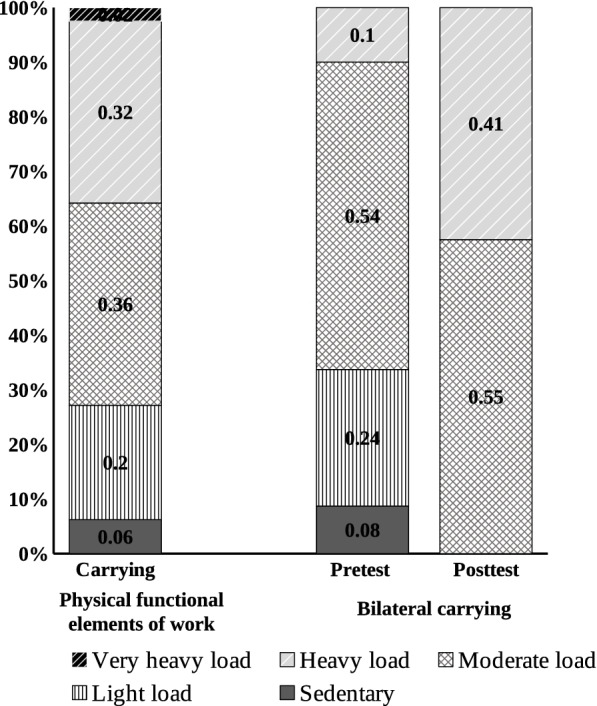
Carrying ability for work and load levels of pre- and post-training bilateral carrying. Note: The numbers do not add up to 100% as some subjects did not complete some of the carrying or lifting tasks

We compared the pre- and post-training scores for carrying and lifting, both required work elements for subjects’ pre-accident jobs, and found that most participants regained their physical capacities in these two elements after work hardening training to a degree that could almost meet their job requirements. In terms of the carrying ability at the workplace, about 26.20% of the participants had a sedentary (6.00%) to light load (20.20%) before accident, and about 70.20% had a moderate (35.70%), heavy (32.10%) or very heavy load (2.40%) before accident. The pre-training results of bilateral carrying showed that after injury, 32.10% of participants were capable of a sedentary (8.30%) or light load (23.80%), and 63.10% of a moderate (53.60%), heavy (9.50%) or very heavy (0%) load. After work hardening training, the bilateral carrying test showed that all participants were capable of at least a moderate load, including 54.80% of a moderate load and 40.50% of a heavy load (Fig. 1).

**Fig. 2:**
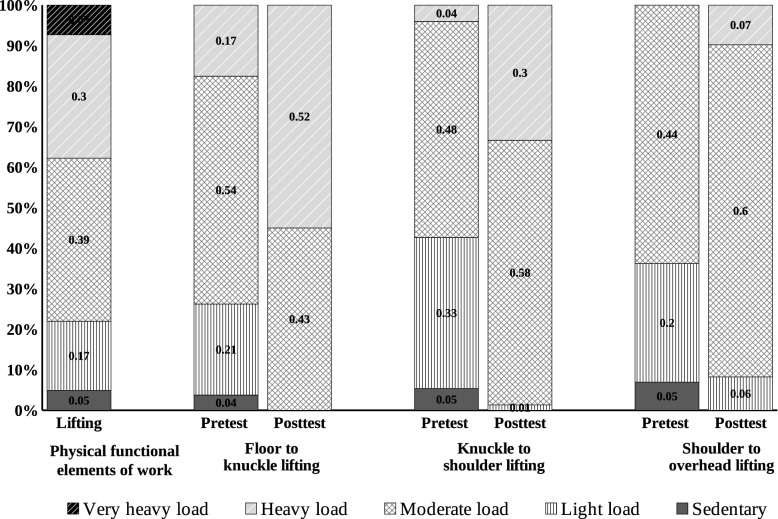
Lifting ability for work and load levels of pre- and post-training bilateral lifting. Note: The numbers do not add up to 100% as some subjects did not complete some of the carrying or lifting tasks

For lifting ability, about 21.50% of the participants were sedentary (4.80%) or had a light load (16.7%) and 76.20% had a moderate (39.30%), heavy (29.80%) or very heavy (7.10%) load required for their work before accident. After accident, 25.00% of the participants were only capable of a sedentary (3.60%) or light load (21.40%) and 70.30% were capable of a moderate (53.60%), heavy (16.70%) or very heavy (0%) load for bilateral floor-to-knuckle lifting. After work hardening training, the bilateral testing showed that all participants were able to lift at least a moderate load, including 42.90% for a moderate and 52.40% for a heavy load. There results indicated an improvement in the bilateral carrying and lifting following work hardening, as shown in Fig. 2.

In the unadjusted models shown in Table 3, the strength subtests were explored for their association with RTW based on the results before and after work hardening training as well as the pre-post training differences. We found that for every additional kilogram in the pre-training bilateral carrying ability, the probability of RTW increased (crude odds ratio [OR] = 1.12, 95% confidence interval [CI] = 1.01–1.24, *p* = 0.027). There were no statistically significant differences observed in the other seven subtests. As bilateral carrying was the only strength test significantly associated with RTW, we then adjusted that model as described in the Methods section, adding covariates to the model sequentially. After adjustment for the basic demographic factors, increased association was observed (adjusted OR = 1.15, 95%CI = 1.01–1.31, *p* = 0.039), which further increased with inclusion of other pre-accident physical functional elements of work (adjusted OR = 1.27, 95%CI = 1.04–1.54, *p* = 0.02). In the fully adjusted model, the odds of successful RTW increased by 27% for every additional kilogram in the pre-training bilateral carrying ability, suggesting that the higher the bilateral carrying capacity of an individual before work hardening training, the easier to RTW (Table 4).

**Table 3:** Relationship between strength categories and return to work

Variables		Preintervention	Postintervention	pre-and-post difference
		OR (95%CI)	OR (95%CI)	OR (95%CI)
**Carrying**	Bilateral carrying	1.12 (1.01–1.24)^*^	1.07 (0.98–1.17	0.95 (0.87–1.05)
**Lifting**	Floor to knuckle lifting	1.14 (0.98–1.34)	0.94 (0.78–1.13)	0.88 (0.75–1.04)
	Knuckle to shoulder lifting	1.17 (0.77–1.76)	1.20 (0.83–1.73)	1.05 (0.67–1.65)
	Shoulder to overhead lifting	0.77 (0.48–1.23)	0.91 (0.66–1.25)	1.25 (0.78–2.01)
**Power grip**	Power grip-left	1.01 (0.96–1.07)	1.05 (0.97–1.14)	1.08 (0.94–1.24)
	Power grip-right	1.01 (0.97–1.05)	0.99 (0.93–1.05)	1.03 (0.90–1.18)
**Lateral pinch**	Lateral pinch-left	0.97 (0.75–1.26)	0.95 (0.66–1.36)	1.03 (0.67–1.58)
	Lateral pinch-right	1.08 (0.85–1.37)	1.11 (0.82–1.48)	0.97 (0.60–1.57)

^*^ *p* < 0.05

*CI,* confidence interval; *OR*, odds ratio

**Table 4:** Multivariate logistic regression analysis of pre-training strength subtest to predict return-to-work with different models

Variables	Model 1^a^	Model 2^b^	Model 3^c^
**Pre-training**			
**Bilateral carrying**	1.12 (1.01–1.24)^*^	1.15 (1.01–1.31)^*^	1.27 (1.04–1.54)^*^
**Demographic characteristics**			
**Age (years)**	–	0.98 (0.91–1.04)	0.98 (0.89–1.09)
**Sex**			
Female vs. male	–	1.79 (0.35–9.09)	13.56 (0.70–265.37)
**Marital status**			
Married vs. unmarried	–	1.31 (0.23–7.46)	2.05 (0.17–24.36)
**Education level**			
High school or less vs. university and above	–	2.07 (0.39–11.11)	8.58 (0.73–101.36)
**Injury site**			
Upper limb vs. Other	–	0.15 (0.01–1.54)	0.67 (0.03–16.65)
Lower limb vs. Other	–	0.06 (0.004–0.80)	0.08 (0.002–2.97)
**Physical functional elements**			
**Pain**			
Yes vs. no	–	–	10.19 (0.71–145.55)
**Carrying**			
Moderate load vs. Mild load	–	–	0.05 (0.001–3.05)
**Lifting**			
Moderate load vs. Mild load	–	–	59.62 (0.92–3869.37)
**Climbing**			
Moderate load vs. Mild load	–	–	3.28 (0.27–40.32)
**Stooping-crouching**			
Moderate load vs. Mild load	–	–	0.28 (0.03–2.61)
**Repetitive sitting-standing**			
Moderate load vs. Mild load	–	–	2.36 (0.32–17.51)
**Walking**			
Moderate load vs. Mild load	–	–	0.09 (0.01–1.58)

*Note*. Physical functional elements pertain to pre-accident job requirements

Physical functional elements load classification: Mild load, including sedentary and light loads; moderate load, including moderate, heavy, and very heavy loads

^*^ *P* < 0.05

^a^ Model 1: pre-training bilateral carrying ability

^b^ Model 2: pre-training bilateral carrying ability plus demographic characteristics

^c^ Model 3: fully adjusted model, including pre-training bilateral carrying ability, demographic characteristics and pre-accident physical functional elements of work

*AOR*, adjusted odds ratio; *CI,* confidence interval

## Discussion

Work hardening mainly provides training to increase muscle strength, including the traditional resistance training and work-related functional resistance training. The combination of these two training modalities offers a great boost to one’s physical capacity [24]. In this study, the participants had substantial improvement in the moderate or more loads of bilateral carrying and bilateral floor-to-knuckle lifting subtests after the training compared to the pre-training results. More importantly, a higher load in the bilateral carrying subtest before the training was associated with higher odds of RTW.

The two strength subtests of bilateral carrying and floor to knuckle lifting showed that all participants were improved to be at least capable of a moderate load after work hardening training, indicating that the participants had increased carrying and lifting ability. According to the biomechanical principle of the lever, the shorter the resistance arm, the lesser force needed (Fig. S1) [25], and according to the length-tension relationship in the exercise physiology, the magnitude of a force depends on the length, speed and tension of the muscle (Fig. S2) [26]. Therefore, the weight to be carried or lifted is closely associated with the length of the muscles involved. Compared to bilateral knuckle-to-shoulder lifting and bilateral shoulder-to-overhead lifting, bilateral carrying and bilateral floor-to-knuckle lifting are less demanding and are easier action modalities, and they have also been shown to have the greatest improvement after the work hardening training [25–27].

There are several possible reasons why the pre-training bilateral carrying ability predicted a higher rate of RTW. A better capacity before training indicates better recovery from the injury, hence a better chance to RTW. From the biomechanical perspective, only bilateral carrying requires walking, and when one foot swings forward, the other must support the entire weight of the body, generating a moment of single-limb support (SLS) [28–30]. In order to hold the body steadily with one foot, one has to call for the muscles on his/her legs and torso, such as the gluteus medius, tensor fasciae latae and quadriceps femoris, and according to some studies, the more strength provided by the leg and torso muscles, the better chance to RTW [31, 32]. Compared to the other strength subtests, bilateral carrying requires not only the upper limbs but also the lower limbs to be able to bear weight. A better result in the pre-training bilateral carrying subtest represents better strength in bilateral training of the subject, which can be translated into a less serious injury to the subject, hence a better chance for the subject’s RTW. Our results showed no associations between post-training results on the strength subtests and successful RTW. Multiple factors could account for this finding, including the degree of subjects’ recovery, presence of chronic pain, and non-injury related factors impacting RTW [14]. The subjects in this study were from a pool of workers receiving government-subsidized rehabilitation. The rehabilitation lasted for 2 months, including 48 hours of work-hardening training. Once a subject finished the 48-hour training, a post-training evaluation was performed immediately. The subjects had improved physical capacity after the training primarily due to neural adaptations, while there was little material changes in the physical health of muscles [33–35], and a substantial proportion of the subjects had not reached the physical capacity required for their pre-injury work or had their pains resolved by the end of the training [12, 36]. According to Gibson et al. [37], the evaluation of whether an injured athlete can return to play depends primarily on his/her pre-injury level of activity and full capacity. Therefore, the strength subtest explored in this study was “one-time” capacity of the subjects, similar to the one-repetition maximum (1RM) in the resistance training. However, RTW requires approximately 8-hour work every day, and muscle endurance must be considered.

In this study, RTW was defined as going back to the pre-accident position. This study found that the higher weight successfully handled in the pre-training bilateral carrying subtest, the easier to RTW. Similarly, Gouttebarge et al. also found that bilateral carrying predicted the probability of RTW and the future work disability [16]. Meanwhile, other studies showed that the bilateral lifting subtests were associated with RTW [9, 14]. For example, Gross and Battié found that the knuckle-to-overhead lifting predicted RTW, and that the better bilateral floor-to-knuckle lifting, the better chance to return to the original position [14]. However, the injury sites of participants and RTW were defined differently in their study compared to the present study, and most previous studies defined RTW simply as working again [8, 9, 14, 16]. It has also been found that the longer one stays out of work, the less possible for him/her to go back to work [9]. In summary, there is evidence for both bilateral carrying and bilateral lifting to predict RTW, but a consistent conclusion is yet to reach due to different injury types, RTW definitions and the duration of non-working period.

This study was limited by the small number of participants, which is why we grouped the five load grades of the six strength subtests into two categories. In addition, although evidence supports the importance of work-hardening [25–27], we did not find associations between any post-training strength subtest result and successful RTW. Other factors necessary for successful RTW that require additional investigation include resolution of chronic pain and recovery of muscle endurance. Practical FCE and reporting are lengthy and time-consuming for healthcare professionals. As this study revealed, the heavier the load handled in pre-training bilateral carrying, the more likely it was for participants to RTW. It is therefore recommended that clinical healthcare professionals with limited time use strength subtests, especially bilateral carrying, to quickly determine the physical capacity of a patient and the possibility of RTW, and provide appropriate RTW recommendations and training. If evaluation shows a good load in the pre-training bilateral carrying but the patient fails to RTW for a long time, further investigation into psychological and social factors may be necessary.

## Conclusion

Workers with occupational accidents are prone to physical capacity decline and consequently work incapacity. While FCE can provide insight into the physical capacity of injured workers, our findings suggest that the bilateral carrying strength subtest is a particularly promising predictor of RTW. Therefore, clinical healthcare professionals, when occupied and busy, can consider prioritizing this subtest to preliminarily determine the physical capacity of the individual, the probability of RTW, and offer further training and RTW recommendations.

## Supplementary Information

**Additional file 1: Table S1.** Description of physical functional elements of work. **Table S2.** Description of eight strength subtests. **Fig. S1.** Schematic diagram of the Chaffin and Andersson model. **Fig. S2.** Length-tension relationship.

## Data Availability

The data that support the findings of this study are available from the corresponding author, [Pei-Ling Tang], upon reasonable request.

## Abbreviations

CI: Confidence interval
DOT: Dictionary of Occupational Titles
FCE: Functional capacity evaluation
ICF: Informed Consent Form
IRB: Institutional Review Board
ROM: Range of motion
RTW: Return to work
MMT: Manual muscle testing
SD: Standard deviation
SLS: Single-limb support
US: United States
WMSDs: Work-related musculoskeletal disorders
