# Supplementary material for: Does category of strength predict return-to-work after occupational injury?
Source: BMC Public Health. 2022 Aug 2;22:1472. doi: 10.1186/s12889-022-13817-2 (PMC9344704; doi:10.1186/s12889-022-13817-2)
Supplement: Supplementary file 1 — Additional file 1: Table S1. Description of physical functional elements of work. Table S2. Description of eight strength subtests. Fig. S1. Schematic diagram of the Chaffin and Andersson model. Fig. S2. Length-tension relationship. [file 12889_2022_13817_MOESM1_ESM.docx]

**Additional file 1**

***Does category of strength predict return-to-work after occupational injury?***

*Authors: Chia-Lin Yang, Yan-Ru Yin, Chuan-Man Chu, Pei-Ling Tang*

**Table of Contents**

**Table S1.** Description of physical functional elements of work

**Table S2.** Description of eight strength subtests

**Figure S1.** Schematic diagram of the Chaffin and Andersson model

**Figure S2.** Length-tension relationship

| **Item** | **Definition** | | **Types of loads** | | | | |
| --- | --- | --- | --- | --- | --- | --- | --- |
|  |  |  | Sedentary | Light load | Moderate  load | Heavy  load | Very heavy  load |
| **Lifting/**(kg) | Raising or lowering an object from one height to another. | Constantly | 0 | 4.5 | 9.1 | 22.7 | 22.7 |
|  |  | Occasionally | 4.5 | 9.1 | 22.7 | 45.5 | 45.5+ |
| **Carrying/**(kg) | Horizontal transport of an object using hands, arms, or shoulders. | Constantly | 0 | 6.8 | 11.4 | 22.7 | 34.1 |
|  |  | Occasionally | 4.5 | 9.1 | 22.7 | 45.5 | 45.5+ |
| **Climbing** | Traveling vertical distance using feet, legs, hands, or arms. | Constantly | N/A | N/A | Stairs | Scaffold | Pilar |
|  |  | Occasionally | Ramp | Stairs | Ladder | Pilar | Rope |
| **Stooping-Crouching/(**times/hour) | Forward bending at the waist- forward and downward bending with flexion at both waist and knees. | | 0 | 15 | 30 | 50 | 60+ |
| **Sitting-Standing**/(min) | Maintain seated position- maintaining vertical position on feet without moving about. | | 30 | 45 | 90 | 180/150 | 210/180+ |
| **Walking**/(times/day) | Horizontal motion using feet. | | 1 | 3 | 4 | 5 | 7 |

**Table S1. Description of physical functional elements of work**

Occasionally: activity up to 33 % of the work time; Constantly: activity from 67% to 100% of the work time; Loaded types: Moderately loaded (including sedentary, light load, and moderate load); heavy loaded (including heavy load and very heavy load) [10].

*Kg*, kilogram.

**Table S2.** **Description of eight strength subtests.**

| **Item** | **Definition** | | **Types of loads** | | | | | | |
| --- | --- | --- | --- | --- | --- | --- | --- | --- | --- |
|  |  |  | Sedentary | Light load | Moderate  load | | Heavy  load | | Very heavy  load |
| **Bilateral carrying** | Raise a box with both hands to the waist height, walk forward 5 meters and turn back to the original spot; the weight will be increased 4–5 times gradually during the test. ^*^Scoring methods: the highest weight of carrying (kg). | | 0–4.4 | 4.5–9 | 9.1–22.7 | | 22.8–45.5 | | >45.6 |
| **Floor to knuckle lifting** | Lower a box from the shelf at finger knuckle height to floor, then raise it back to shelf at finger knuckle height again. The weight will be increased 4–5 times gradually during the test. ^*^Scoring methods: the highest weight of lifting (kg). | |  |  |  |  |  |  |  |
| **Knuckle to shoulder lifting** | Raise a box from the shelf at finger knuckle height to the shelf at shoulder height, then lower it back to the shelf at finger knuckle height again. The weight will be increased 4–5 times gradually during the test. ^*^Scoring methods: the highest weight of lifting (kg). | |  |  |  |  |  |  |  |
| **Shoulder to overhead lifting** | Raise a box from the shelf at shoulder height to the shelf at eye height, then lower it back to the shelf at shoulder height again. The weight will be increased 4–5 times gradually during the test. ^*^Scoring methods: the highest weight of lifting (kg). | |  |  |  |  |  |  |  |
|  | | | **Poor** | **Average** | | **Good** | | **Excellent** | |
| **Power grip-left** | Test grip strength with grip strengthener three times. ^*^Scoring methods: average grip strength (kg). | Male | <28.9 | 29.0–37.3 | | 37.4–45.7 | | >45.8 | |
|  |  | Female | <17.4 | 17.5–23.3 | | 23.4–28.7 | | >28.8 | |
| **Power grip-right** |  | Male | <30.9 | 31.0–40.0 | | 40.1–49.2 | | >49.2 | |
|  |  | Female | <18.8 | 18.9–24.7 | | 24.8–30.7 | | >30.8 | |
| **Lateral pinch-left** | Test lateral pinch strength with pinch meter three times. ^*^Scoring methods: average pinch strength (kg). | Male | <6.2 | 6.3–8.3 | | 8.4–10.6 | | >10.7 | |
|  |  | Female | <4.5 | 4.6–6.3 | | 6.4–8.2 | | >8.3 | |
| **Lateral pinch-right** |  | Male | <6.5 | 6.6–9.2 | | 9.3–11.3 | | >11.4 | |
|  |  | Female | <5.2 | 5.3–6.6 | | 6.7–8.6 | | >8.7 | |

*Kg*, kilogram.


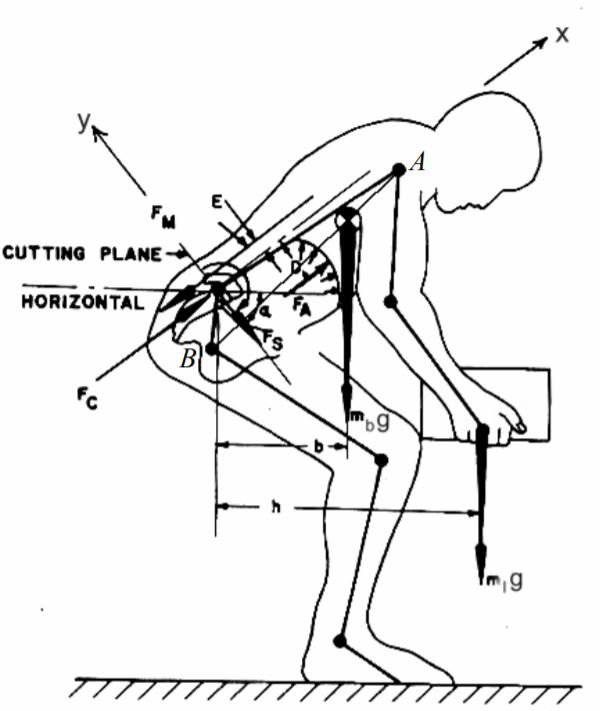


**Figure S1.** Schematic diagram of the Chaffin and Andersson model [25]


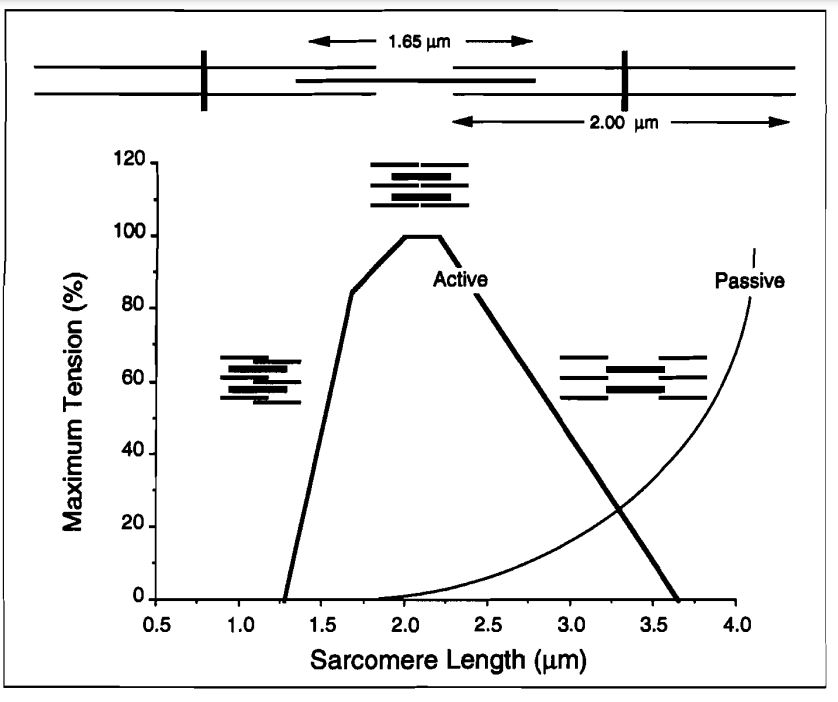


**Figure S2.** Length-tension relationship [26]
